# Housing Stakeholder Preferences for the “Soft” Features of Sustainable and Healthy Housing Design in the UK

**DOI:** 10.3390/ijerph13010111

**Published:** 2016-01-07

**Authors:** Agne Prochorskaite, Chris Couch, Naglis Malys, Vida Maliene

**Affiliations:** 1 Department of the Built Environment, Liverpool John Moores University, Cherie Booth Building, Byrom Street, Liverpool L3 3AF, UK; a.prochorskaite@2009.ljmu.ac.uk; 2 School of Environmental Sciences, University of Liverpool, Jane Herdman Building, Liverpool L69 3GP, UK; C.Couch@liverpool.ac.uk; 3 School of Life Sciences, Centre for Biomolecular Science, The University of Nottingham, Nottingham NG7 2RD, UK; Naglis.Malys@nottingham.ac.uk; 4 Institute of Land Management and Geomatics, Faculty of Water and Land Management, Aleksandras Stulginskis University, Universiteto 10, Akademija, Kaunas LT-53361, Lithuania

**Keywords:** sustainable housing, quality of life, health and well-being, housing stakeholder, preference survey, soft feature

## Abstract

It is widely recognised that the quantity and sustainability of new homes in the UK need to increase. However, it is important that sustainable housing is regarded holistically, and not merely in environmental terms, and incorporates elements that enhance the quality of life, health and well-being of its users. This paper focuses on the “soft” features of sustainable housing, that is, the non-technological components of sustainable housing and neighbourhood design that can impact occupants’ health and well-being. Aims of the study are to ascertain the relative level of importance that key housing stakeholders attach to these features and to investigate whether the opinions of housing users and housing providers are aligned with regards to their importance. An online survey was carried out to gauge the level of importance that the key stakeholders, such as housing users, local authorities, housing associations, and developers (*n* = 235), attach to these features. Results revealed that while suitable indoor space was the feature regarded as most important by all stakeholders, there were also a number of disparities in opinion between housing users and housing providers (and among the different types of providers). This implies a scope for initiatives to achieve a better alignment between housing users and providers.

## 1. Introduction

United Kingdom (UK) today is facing the challenge of having to build around 300,000 new homes each year to address its current housing shortage and meet the needs of its population [1,2]. In light of this challenge, the design of new housing has become a highly pertinent issue as there is widespread recognition that the quality and sustainability of new build homes need to improve [2,3]. This means that the understanding, development and provision of “sustainable housing” need to go beyond environmental efficiency measures and embrace design features that improve quality of life, and in particular, the health and well-being of its residents [4,5].

Despite the growth of interest in the sustainability of urban environments over the last few decades, there has also been criticism that not enough has been done to conceptualise the meaning of “sustainable housing” [6,7]. As a consequence, the term continues to be widely used to describe housing with a lower environmental impact (such as housing with enhanced energy efficiency features), while other sustainability aspects, especially social (including health and well-being), economic and cultural factors, are largely overlooked [4,5,8,9].

However, a dwelling with enhanced environmental performance cannot be regarded as sustainable if the housing users’ quality of life is negatively impacted by factors such as layout restrictions, lack of natural lighting or by the need to move to a different home if their circumstances change. While safeguarding the environment is obviously an important goal, Chiu [10] argues that the primary aim of a sustainable housing development should be to meet the needs of the people rather than to preserve the environment.

One approach that can be used to define this concept is to apply the basic principle of sustainable development to housing. While, admittedly, the exact definition of sustainable development itself is not always agreed upon, there is a broad consensus that the fundamental aim of sustainable housing should be about enhancing the quality of human life for current and future generations [4,11]. Indeed, a strategy report, Caring for the Earth, published in 1991 by the World Conservation Union (IUCN), United Nations Environment Programme (UNEP) and World Wide Fund For Nature (WWF), defines the concept of sustainable development as a process that improves “the quality of human life while living within the carrying capacity of supporting ecosystems” [12] (p. 10).

Taking the idea that quality of human life is at the core of the sustainable development concept and applying it to housing implies that the fundamental aim of “sustainable housing” should be about enhancing the quality of human life. In other words, the design of sustainable housing should ensure a better quality of life for current residents as well as future generations taking into account wider social, environmental and economic aspects [6,8,13].

Health and well-being are fundamental to quality of life, and, therefore, inextricably linked to sustainable development. Of the 27 guiding principles for sustainable development set out by the Rio Declaration on Environment and Development during the 1992 Earth Summit, the first one makes this connection:

“Principle 1: Human beings are at the centre of concerns for sustainable development. They are entitled to a healthy and productive life in harmony with nature” [14].

The same interconnectedness can be extrapolated to health and sustainable housing as the two agendas are ultimately about enhancing the quality of human life within the wider socio-economic and environmental contexts. As Barton and colleagues [15] point out, health can be regarded as “the touchstone for sustainability” (p. 15) given that the fundamental principles provide common ground for both, as healthy neighbourhoods will also likely be sustainable neighbourhoods.

This study focuses on the “soft” features of sustainable housing, that is, the non-technological elements of housing and neighbourhood design that can impact the health and well-being of residents as well as the satisfaction with their homes. While there is much discussion in literature about the various “soft” housing design features that can impact on health and well-being, existing studies typically focus on one or a small number of such features. This paper presents them as part of the broader sustainable and healthy housing framework in order to ascertain the level of importance that key housing stakeholders attach to these features and to determine whether the opinions of housing users and housing providers are aligned with regards to their importance. We investigate how the opinions of housing users and housing providers including local authorities, housing associations, and developers are aligned. By doing so, it reveals some of the apparent differences in priorities that housing users and housing providers attach to these features (and indeed differences between social housing and private sector providers). These differences between housing user and housing provider priorities have important implications for practice, and suggest that there is scope for initiatives to achieve a better alignment of opinion regarding these features.

## 2. Methodology

### 2.1. Data Collection

#### 2.1.1. Sample

Recruitment of respondents to the survey began with the development of a database of the four housing stakeholder groups within the geographical area of the study. Invitation emails were sent to contacts in each group and respondents were asked to forward the link to their contacts. The “housing user” group consisted of community groups and residents associations supplemented with members of staff from a West Midlands based University as well as social media (LinkedIn) contacts. For all of the housing provider groups, a list of relevant organisations within the region was collated (*i.e.*, all local authorities (51), housing associations (80) and private sector developer companies (49)). Within these organisations, 430 individuals in high level management, housing design or development roles were contacted to fill in the questionnaire. In total, 235 respondents completed the survey, of which 123 were “housing users” and 112 were housing developers. While for housing users, the response rate is unavailable due to the snowball sampling approach, for housing providers this represents a 26% response rate (48 from housing associations, 34 from local authorities and 30 from private sector housebuilding companies).

#### 2.1.2. Measures

To investigate the level of importance that stakeholders attach to the 11 “soft” features of housing and neighbourhood design, we developed an online questionnaire utilising a 5-point Likert rating scale to represent the level of importance. Respondents were asked how important each of the 11 features were to them and to rate each one with a score ranging from “1” (= “not at all important”) to “5” (= “extremely important”) reflecting their opinion. The survey was administered online using the Bristol Online Survey programme to four groups of housing stakeholders (“housing users”, housing associations, local authorities and private sector developers) in the West Midlands and parts of the North West regions of England. Because of the subjective nature of the research (*i.e.*, opinions of importance), the questionnaire was limited to one geographical area of the UK to maintain any social, historical, economic and cultural factors that might influence opinion constants. Respondents were invited to fill in the survey via a personalised cover email which contained the URL link for the questionnaire. The link was available from August 2013 to January 2014.

### 2.2. Statistical Analyses

To compare the level of importance that the different groups attached to each of the “soft” features, two types of data analyses were conducted using SPSS (v21): Descriptive statistics (measures of central tendency) and non-parametric tests (Mann–Whitney U and Kruskal–Wallis H).

While it is acknowledged that using measures of central tendency implies interpreting the Likert rating scale as having interval qualities and the debate around the use of such techniques on ordinal data is recognised (e.g., discussions by [16]), it was deemed in this case to be a useful and necessary simplification of rating scores and an approach that is commonly used in interpreting preference surveys [17].

Non-parametric procedures were chosen because normal distribution of data could not be assumed following the Kolmogorov–Smirnov (K–S) test to assess for normal distribution of scores. K–S significance values for all variables were well below 0.05 significance level (*p* = 0.000), indicating that the distribution of scores in all stakeholder samples significantly deviates from normal distribution.

## 3. Results

### 3.1. “Soft” Features of Sustainable Housing and Neighbourhood Design

**Figure 1:**
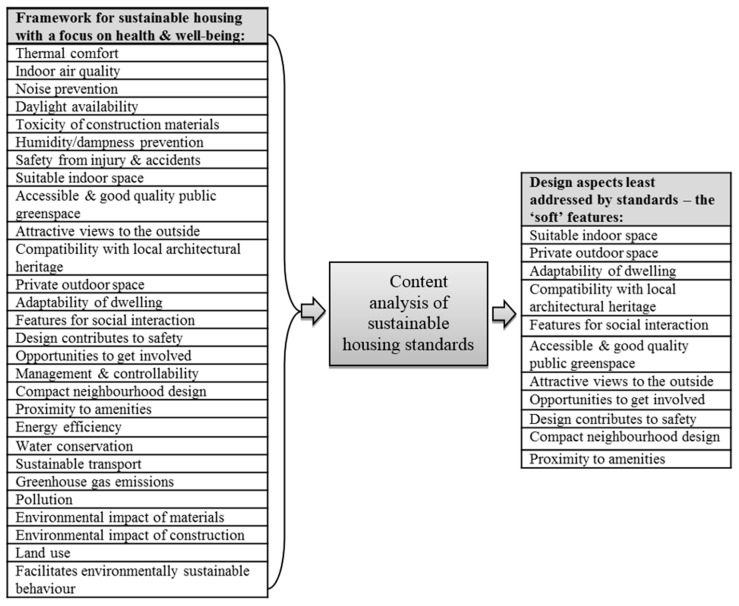
Summary schematic of the process used to identify 11 “soft” features of sustainable housing and neighbourhood design.

The “soft” features of sustainable housing and neighbourhood design for use in the survey were identified through a two stage process (Figure 1) [18]. Given that a framework based approach is a more appropriate way to define and discuss the concept of sustainable housing [7,9], a broad framework for sustainable housing with an emphasis on health and well-being was developed. An extensive review was carried out of the sustainable housing literature as well as housing and health research, and, from this review, 28 housing characteristics were identified as components of this framework given their importance to healthy and sustainable housing.

The second stage involved a content analysis of eight sustainable housing standards. These eight standards from the UK and abroad (The Code for Sustainable Homes (UK), BREEAM Domestic Refurbishment (UK); R-2000 (Canada), High Quality Environmental standard (France), Comprehensive Assessment System for Built Environment Efficiency (Japan), SB Tool (International), GreenStar (Australia), and Leadership in Energy & Environmental Design (US)) were used to broadly represent industry’s best practice, and each of the 28 components of the sustainable and healthy housing framework were reviewed against these standards to ascertain the level to which they have been addressed. The 11 aspects that received least coverage (Figure 1) are the focus of the remainder of the study. These were labelled as the “soft” features of sustainable housing because they are all non-technological elements of housing and neighbourhood design that can have an indirect, but nevertheless important impact on the health and well-being of residents and the satisfaction with their homes [19].

The 11 “soft” features, also described in more detail in [19], and why they are important factors to the quality of life, particularly to health and well-being, are as follows:

***Suitable indoor space (F1)*:** Research evidence suggests that inadequate space in the home can negatively affect educational outcomes of children, family relationships as well as the well-being of individuals [20]. These effects can arise through lack of space to socialise with household members and guests, lack of privacy (e.g., inability to work, study and relax in a quiet environment), through the impact on diet and nutrition due to inadequate space for food preparation, as well as limited opportunities to pursue certain hobbies or keep a pet [2,21,22,23].

***Private outdoor space (F2)*:** Benefits of gardens tend to be dependent on cultural values; however, depending on their size, private gardens can offer opportunities for creativity and self-expression, exercise and restoration from stress, personal satisfaction from sense of achievement, relaxation, socialising, and food production [24,25].

***Adaptability of the dwelling (F3)*:** Internal space and layout should be designed in a way that would allow areas of the dwelling to be adapted, converted or extended reflecting the changing circumstances of the occupants and ensuring a greater lifespan of the home. The inability to adapt a home can have a negative impact on the quality of life due to crowding, lack of privacy, reduced mobility and the generally reduced ability to fully participate in day-to-day activities such as food preparation, reducing comfort and pride in one’s home [21]. Such restrictions will be particularly felt by those who cannot afford to move. However, those who are able to move home to meet their space needs may experience indirect negative consequences such as disrupted social relationships, greater financial strain and the stress of moving.

***Compatibility with local architectural heritage (F4)*:** The exterior design of housing is not merely a personal matter of aesthetic preference. As a component of culture [13], housing is a public good that becomes a part of the landscape and the heritage of a nation [26]. By helping people to identify with the past and their heritage, new housing developments that are sensitive to the local vernacular can foster a sense of belonging and help enhance the quality of life [15,27].

***Features in the neighbourhood for informal social interaction (F5)*:** There is growing evidence that affirmative social interaction and support can have a number of positive health and well-being effects mental health mood, anxiety and stress levels [28]. While housing and neighbourhood design itself cannot develop social networks, provision of certain features (e.g., seating areas, playgrounds, picnic sites, communal gardens) can facilitate the growth of social interactions and support existing ones. Such features can therefore not only contribute to quality of life and well-being, particularly for the less mobile societal groups such as the elderly [29], but also promote the growth of social capital, thereby supporting the development of strong and sustainable communities [30].

***Accessible and good quality public greenspace (F6)*:** There is a substantial and growing body of research building the evidence base for a positive impact that natural environments have on physical and mental health and well-being (e.g., [31,32]). While the exact links have not been fully elucidated, the positive impacts on health may be through greater active outdoor recreation [33], by providing a restorative function from stress and enhancing positive emotions [34,35] as well as through greater opportunities for social interaction [36]. After a comprehensive review of the evidence, the 2007 Royal Commission on Environmental Pollution report on the Urban Environment concluded that “the evidence is sufficiently strong to warrant amending planning guidance to recognise the health benefits of green space and to build green space into new and existing developments” [37] (p. 47). However, it is important to note that quality and accessibility are crucial factors in mediating this relationship between greenspace and health [31].

***Attractive views to the outside (F7)*:** Bad views from the dwelling was found by the World Health Organisation’s (WHO) Large Analysis and Review of European Housing and Health Status (LARES) study (2002–2003) to be one of the four key housing problems linked to increased prevalence of mental health symptoms (other factors were missing daylight, noise, and inadequate privacy perception) [38]. While it would be unrealistic to expect highly attractive views from all rooms of a dwelling in a typical housing development, there is evidence that views of nature or natural features are associated with a higher sense of well-being and a more positive perception towards the quality of the overall neighbourhood [39].

***Opportunities to get involved (F8)*:** Meaningful community engagement is an important factor in a housing development as it communicates housing users’ concerns and opinions, instills a sense of pride in the neighbourhood, increases the sense of ownership and helps to build social capital [15,40]. It is becoming recognised that developers can support the establishment of community governance structures that would enable residents to participate in the management of the neighbourhood, thereby instilling a greater sense of ownership and pride in the area [2].

***Dwelling and neighbourhood design that contributes to safety from crime (F9)*:** Fear of crime has been linked to health and well-being not only in relation to mental distress [41] but also physical health as body mass index has been found to be higher among residents with lower levels of perception of safety in their neighbourhoods [42]. The perception of safety, fear of crime as well as actual levels of crime can be influenced through housing and neighbourhood quality and design [41,43]. Structural elements that can exacerbate fear of crime include windows that do not close properly, poor boundary design, inadequate lighting and management of public areas and inability to overlook the street from the house [36].

***Compact neighbourhood design (F10)*:** The issue of residential density in a sustainable housing context is complex and contentious. While compact housing developments are often regarded as more sustainable forms of the built environment [15,44], some argue that the extent and nature of benefits associated with higher densities are less clear cut and higher density urban forms may produce trade-offs between the core elements of sustainability [45]. However, proponents of higher density housing argue that previous failures of high density built forms are more the result of underlying social problems and management issues than the residential density factor [44].

***Proximity to amenities (F11)*:** Availability of and easy access to local amenities such as shops, entertainment, healthcare, education and other facilities have been linked to a number of social, economic and health benefits. These include reduced number of car trips (leading to greater physical activity, reduced air pollution and congestion), greater number of casual or planned interactions that encourage the formation and reinforcement of social networks and the sense of local community, as well as a source of local jobs and a benefit to the local economy [15].

### 3.2. Reliability

Intra-class correlation (ICC) was used to assess the degree of agreement among the respondents of each group, which is one of the most commonly used methods for assessing the inter-rater reliability (IRR) for ordinal, interval, and ratio data [46,47]. The ICC value can range from 0 (no agreement) to 1 (complete agreement) and commonly used cut-offs are as follows; the IRR is considered “poor” for ICC values less than 0.40, “fair” for values between 0.40 and 0.59, “good” for values between 0.60 and 0.74 and “excellent” for values between 0.75 and 1.0 (cited in [47]).

Table 1 shows average-measure ICC values for the four stakeholder groups. The IRR is considered to be excellent for all groups as the ICC values are above 0.9. While we are more interested in the “absolute agreement” values, the high consistency values indicate that there is also a high level of agreement in terms of the rank order of the 11 features.

**Table 1:** Average-measures intra-class correlation (ICC) values for each stakeholder group.

Stakeholder Group	Average Measures ICC	
	Consistency	Absolute Agreement
“Housing users”:	0.984	0.981
Housing associations:	0.974	0.969
Local authorities:	0.947	0.939
Developers:	0.968	0.953

### 3.3. Importance Rating Scores

**Figure 2:**
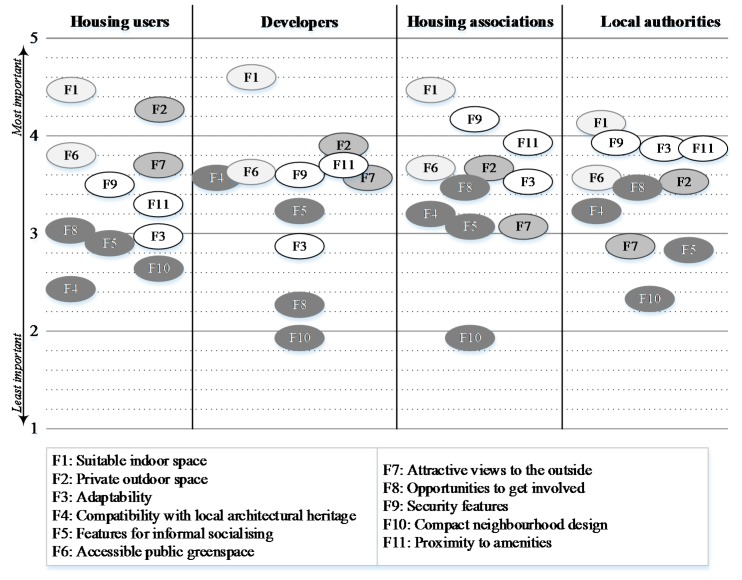
Mean scores of importance for the 11 “soft” features (F1–F11) as rated by the four housing stakeholder groups (from 1 = “not at all important” to 5 = “extremely important”). (NB. The shading of the features is for clarity and does not relate to importance).

The mean scores of importance rated by housing users (*n* = 123), housing associations (*n* = 48), local authorities (*n* = 34) and developers (*n* = 30) were calculated and are shown in Figure 2. Using these scores, the relative importance can be explored from two angles; firstly, there is the resulting average ranking assigned by each stakeholder group for each feature, and secondly, the actual value assigned.

All groups rated indoor space (F1) as the most important feature. Of the four groups, developers gave this feature the highest average score (M = 4.60, SD = 0.5) followed by housing associations (M = 4.48, SD = 0.58), “housing users” (M = 4.47, SD = 0.67), and lastly local authorities (M = 4.15, SD = 0.66). Outdoor space (F2) was rated second in importance by both the “housing users” and developers, although “housing users” gave this feature a higher average score (M = 4.31, SD = 0.91) than did the developers (M = 3.90, SD = 0.80). Outdoor space was scored much lower by housing associations (M = 3.65, SD = 0.67) and local authorities (M = 3.53, SD = 0.71)—placing it in fifth and sixth place, respectively. Both social housing providers allocated security (F9) to the second place, while this criterion came fifth in importance for both “housing users” (M = 3.50, SD = 1.07) and developers (M = 3.60, SD = 0.86).

Greenspace (F6) came third in terms of importance for the “housing users” group (M = 3.80, SD = 0.96). This was rated similarly, fourth in importance, by both housing associations (M = 3.67, SD = 0.86) and developers (M = 3.63, SD = 1.03), and came a little lower in importance, fifth, for the local authority respondents (M = 3.56, SD = 0.86). Interestingly, “housing users” rated attractive views (F7) as fourth in importance (M = 3.73, SD = 0.84) while all housing providers, but particularly social housing providers, marked this much lower in importance; housing associations scored it tenth (M = 3.06, SD = 0.91), local authorities—ninth (M = 2.82, SD = 0.78) and developers scored it seventh (M = 3.57, SD = 0.90). From the mean scores, however, it can be seen that out of the three housing provider groups, developers gave this feature the highest rating and local authorities the lowest.

Another difference in the level of importance that is interesting to note relates to “access to amenities” (F11). “Housing users” placed this feature in the middle place, sixth, with a mean score of 3.33 (SD = 1.03). However, all of the housing providers allocated a higher level of importance to this feature—both housing associations and developers placed it in third place (M = 3.94, SD = 0.73 and M = 3.73, SD = 0.94 respectively) and local authorities marked this in fourth place (M = 3.88, SD = 0.81).

Local authorities differed from all the other stakeholder groups in the relatively high level of importance they allocated to adaptability (F3). Local authority respondents marked adaptability as third in importance with the mean score of 3.88 (SD = 0.95). “Housing users”, on the other hand, assigned this feature to eighth place with a relatively low mean score of 2.98 (SD = 1.12). Similarly, developers placed it ninth with the mean score of 2.83 (SD = 0.99), while housing associations have it slightly higher rating of 3.56 (SD = 0.85) placing it sixth.

No major differences were seen in the ratings given to the compatibility with local architectural heritage feature (F4). Both of the social housing providers placed this feature in the eighth position with housing associations scoring it an average of 3.21 (SD = 0.82) and local authorities, 3.24 (SD = 0.86). “Housing users” scored this lower at 3.04 (SD = 1.11), although placed at a slightly higher ranking of seventh. Among the four groups, developers gave it the highest scoring of 3.56 (SD = 0.97), placing it in sixth position of relative importance.

Features for informal socialising (F5) and opportunities to get involved (F8) were scored low by all groups. F5 received an average score of 2.89 (SD = 1.12) from the “housing users”, placing it in the ninth position, while housing associations placed it in the same position with an average score of 3.06 (SD = 0.81). Local authorities allocated F5 to the tenth position of relative importance with scores of 2.82 (SD = 0.80), while developers marked it slightly higher with 3.23 (SD = 1.10), placing it in eighth position. For opportunities to get involved (F8), both of the social housing providers placed this in the seventh position with housing associations, scoring it 3.48 (SD = 1.15) and local authorities scoring it similarly with 3.47 (SD = 0.83). “Housing users” and developers placed F8 lower, in the tenth position, with the former giving this feature a slightly higher average score of 2.66 (SD = 1.09) than the latter group who scored it an average of 2.27 (SD = 0.91).

Lastly, all groups placed compact neighbourhood design (F10) as the last in the list of relative importance. In terms of the mean scores, “housing users” scored this highest of the four groups at 2.43 (SD = 1.09), followed by local authorities at 2.32 (SD = 0.95), with housing association and developers giving it very similar scores of 1.94 (SD = 0.84) and 1.93 (SD = 0.83), respectively.

### 3.4. Comparing Opinions among Stakeholder Groups

#### 3.4.1. “Housing Users” *versus* Housing Providers

Mann–Whitney U test was carried out on the scores of each feature to determine whether there is a statistically significant difference between the levels of importance allocated by the “housing users” group (*n* = 123) and the housing providers (*n* = 112) (Table 2). Statistically significant differences (*p* < 0.05) were found for seven features; private outdoor space (F2), adaptability (F3), attractive views (F7), opportunities to get involved (F8), security features (F9), compact neighbourhood design (F10) and proximity to amenities (F11).

“Housing users” gave higher scores (indicated by the higher mean ranks) to private outdoor space (F2), attractive views (F7), and compact neighbourhood design (F10). Conversely, housing providers gave higher scores to adaptability (F3), opportunities to get involved (F8), security features (F9), and proximity to amenities (F11). The effect sizes (*r* values) for all seven of the criteria are in the medium range of effect (Following Cohen’s [48] criteria of 0.1 = small effect, 0.3 = medium effect and 0.5 = large effect), ranging from highest for private outdoor space (*r* = 0.41) to lowest for compact neighbourhood design (*r* = 0.27).

#### 3.4.2. Housing Providers

**Figure 3:**
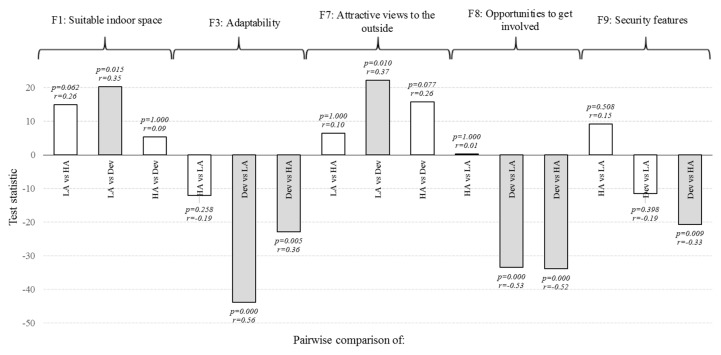
The results for each group pair comparison, including test statistic (difference between mean ranks), the adjusted *p*-values, effect size (*r*) and subsequent decision regarding H_0_ (*grey bar indicates* = *H*_0_ *rejected*). Groups compared: Local authorities (LA), housing associations (HA) and private sector developers (Dev).

The Kruskal–Wallis H test was utilised to explore whether there are any differences in opinion regarding the importance of features among the three housing provider groups (Figure 3). Statistically significant difference (*p* < 0.05) in opinion was found for five features; indoor space (F1), adaptability (F3), attractive views (F7), opportunities to get involved (F8) and security features (F9). *Post hoc* Mann–Whitney U tests were carried out using the adjusted α level of 0.017 (following the Bonferroni procedure to control for Type I error) on pairwise comparisons to identify which housing providers differed in opinion.

**Table 2:** Results of Mann–Whitney U test, including test statistics (*U* value), effect size (*r*), *z*-value, *p*-value and the resulting decision regarding H_0_ (=no difference in the distribution of score rankings).

Criterion	Stakeholder Group	Mean Rank	Median	*U*-Value	Effect Size (*r*)	*z*-Value	*p*-Value	Decision
F1: Suitable indoor space	Housing provider	113.55	4	6390	−0.07	−1.077	0.281	Retain H_0_
	Housing user	122.05	5					
F2: Private outdoor space	Housing provider	90.50	4	3808	−0.41	−6.276	0.000	Reject H_0_
	Housing user	143.04	5					
F3: Adaptability	Housing provider	132.34	3	5282	−0.21	−3.206	0.001	Reject H_0_
	Housing user	104.94	3					
F4: Compatibility with architectural heritage	Housing provider	126.08	3	5984	−0.12	−1.821	0.069	Retain H_0_
	Housing user	110.65	3					
F5: Features for informal socialising	Housing provider	122.82	3	6349	−0.07	−1.091	0.275	Retain H_0_
	Housing user	113.61	3					
F6: Accessible public greenspace	Housing provider	110.76	4	6078	−0.11	−1.638	0.101	Retain H_0_
	Housing user	124.59	4					
F7: Attractive views to outside	Housing provider	95.64	3	4384	−0.33	−5.069	0.000	Reject H_0_
	Housing user	138.36	4					
F8: Opportunities to get involved	Housing provider	133.27	3	5178	−0.22	−3.401	0.001	Reject H_0_
	Housing user	104.09	3					
F9: Security features	Housing provider	132.07	4	5313	−0.21	−3.190	0.001	Reject H_0_
	Housing user	105.19	4					
F10: Compact neighbourhood	Housing provider	106.46	2	5595	−0.17	−2.594	0.009	Reject H_0_
	Housing user	128.51	2					
F11: Proximity to amenities	Housing provider	136.36	4	4832	−0.27	−4.162	0.000	Reject H_0_
	Housing user	101.28	3					

For indoor space (F1), statistically significant differences were found between local authorities and developers (*p* = 0.015), with the latter giving higher scores (mean rank 65.0) than local authorities (mean rank 44.6). For adaptability (F3), statistically significant differences were found between developers and local authority respondents (*p* = 0.000) as well as between developers and housing associations (*p* = 0.005). In both comparisons, respondents in housing associations (mean rank 58.97) and local authorities (mean rank 70.94) gave higher scores than those in the developer group (mean rank 35.18). For the attractive views feature (F7), a statistically significant difference was found between local authority and developer groups (*p* = 0.010), with developers giving higher scores (mean rank 70.05) than local authorities (mean rank 47.75). Regarding opportunities to get involved (F8), a statistically significant difference was found between developers and local authorities (*p* = 0.000) as well as developers and housing associations (*p* = 0.000): In both comparisons, housing association (mean rank 65.68) and local authority (mean rank 65.28) respondents scored F8 higher than developers (mean rank 31.87). For security features (F9), a statistically significant difference was found between developer and housing association groups (*p* = 0.009), with the latter giving higher scores (mean rank 64.84) than developers (mean rank 44.20). The effect size ranged from medium (developers *vs.* local authority on security features, *r* = −0.33) to large (developers *vs.* local authority on adaptability, *r* = −0.56).

## 4. Discussion

The aim of this paper was to gauge the level of relative importance that key housing stakeholders attach to the 11 “soft” features of housing and neighbourhood and to explore how opinions differ between housing users and housing providers. While some features appeared to be generally more important than others, a number of interesting differences can be discerned in the opinions of the four housing stakeholders.

Indoor space was rated highest in relative importance by all stakeholder groups and no statistically significant differences were observed between the scores of housing users and the housing provider groups. This importance of indoor space to occupants is in line with other consumer preference surveys, such as the research carried out by the Commission for Architecture and the Built Environment (CABE) and Royal Institute of British Architects (RIBA) (e.g., [20,21,49,50]). For instance, 80% of respondents in a 2013 RIBA survey stated that they would be more likely to choose a home that met minimum space standards over standards relating to energy, security and access [50].

This is particularly pertinent given the relatively small average indoor area of new housing in the UK. In 2011, a RIBA study found that an average three bedroom home in England was 88 sqm—92% of the minimum Greater London Authority space standards, which the study was using as a benchmark. The average size appeared to be slightly better in the 2012 English Housing Survey [51], which calculated the average total floor area of new homes (built since 2002) to be 96 m^2^. This higher average size of new homes was boosted by some very large dwellings. Perhaps a more revealing statistic from the Housing Survey was that a higher proportion (44%) of new builds had a useable floor space under 69 m^2^, compared with 35% of older dwellings.

Concern over the decrease of internal space of new homes has been voiced ever since the withdrawal of the Parker Morris standards in 1980, which had stipulated minimum house sizes for publically funded homes [23]. In an attempt to remedy this, the UK Government announced in 2014 the introduction of a new national minimum space standard for new homes in England following its Housing Standards Review [52]. However, adoption of this national space standard will be voluntary as an optional planning condition whereby local authorities will be required to justify the local viability of the standard before it is adopted. In light of the space standard’s reliance on local authorities for implementation, it is interesting that this survey found a statistically significant difference between the importance scores for internal space between local authorities and private sector developers, with the latter giving higher rating to this feature than local authorities.

For housing users and private sector developers, private outdoor space came second in importance after indoor space—much higher in ranking than allocated by the two social housing providers. However statistical tests of the importance scores revealed that this was a feature over which housing users and housing providers differed most—with the former giving higher ratings than the latter. Despite the relatively high ratings given by the developers than social housing providers to outdoor space, Kruskal–Wallis H test revealed no statistically significant differences among the three housing provider groups.

The importance of private outdoor space to housing consumers supports findings of previous research [44,49,53]. However the apparent discrepancy between the opinions of housing users and housing providers in this survey is interesting. Housing providers are under increasing pressure to build to higher densities, effectively limiting the provision of adequately sized private gardens—it would be interesting to ascertain through further research whether there is an element of downplaying the relative importance of private outdoor space by the housing providers, particularly among housing associations and local authorities.

In addition, an interesting comparison can be made here with the importance attached to the availability of good quality and accessible public greenspace. In this study, housing providers rated public greenspace and private outdoor space similarly in terms of importance, while housing users scored the former as slightly less important than the latter. Greenspace can afford similar benefits as an outdoor private space, such as access to nature and active recreation, and as such may be treated by housing providers as a substitute for larger gardens [54]. However, a public greenspace cannot fully substitute a garden, the privacy of which enables many to view and treat it as an important extension of main living and social areas of the home [49].

Another feature that appeared to be much more important to housing users than to housing providers was the presence of attractive views from the dwelling. It is difficult to link this to findings in other housing consumer surveys in the UK context as these tend to focus on window design more from light availability or security perspectives rather than orientation for views. For instance, RIBA’s research found that in dense developments, windows overlooking other homes can intrude on people’s sense of privacy [49]. However, the type of view from a dwelling appears to be an important feature to occupants that warrants greater attention in housing consumer surveys. Bad view from the dwelling was one of the four key housing problems that were linked to increased prevalence of mental health symptoms [38]. Previous research showed that private outdoor space, public greenspace and attractive views from the dwelling, all benefited health [32,33,35,38,39].

Three features were generally perceived more important by housing providers than by housing users: House and neighbourhood design that contributes to safety from crime was one such feature ranked higher by social housing providers than by housing users (or private sector developers). This relatively low ranking awarded by housing users has also been found in other surveys where it has been suggested that this may be due to participants regarding security as their own responsibility rather than that of those designing or developing homes [49].

A similar situation was observed for dwelling adaptability. There has been much discourse over the values and benefits of adaptable homes [23], so much so that from 2010, “Lifetime homes” has become the only mandatory element under the “Health and Well-being” category of the Code for Sustainable Homes (at level 4) [55]. Interestingly, the importance of adaptability was generally scored much lower by housing users than by housing providers, particularly local authorities and housing associations. This may be due to lack of awareness of the benefits of an adaptable dwelling: As few properties are amenable to easy and cost effective adaptation, people are more accustomed to moving home as their circumstances change. The low scoring by private sector developers, however, is unsurprising as demand for new and greater range of homes is favourable to business turnover.

Proximity to amenities was the third feature that housing providers ranked higher in importance than did the housing users group. This relatively low rating of importance has also been found by earlier surveys. For instance, earlier CABE research found that more people were willing to forgo amenities for the right home than *vice versa* [44].

The four features that were ranked lowest in importance by all stakeholders, and with little variations in opinions among the groups, were: Features for informal socialising, compatibility with local architectural heritage, opportunities to get involved, and, lastly, a more compact (higher density) neighbourhood design. Arguably, it is no coincidence that these four are also the most complex and potentially multifaceted of the 11 features.

## 5. Recommendations

The findings of this paper suggest there is scope for initiatives to achieve a better alignment of opinions regarding these features. For instance, housing users regarded private outdoor space and attractive views as much more important than did the housing providers. Having health implications, these features may need greater attention in the design briefs of the latter. Conversely, housing providers (especially social housing providers) viewed adaptability, design features that enhance security, and access to amenities as more important than did the housing users. This suggests that educational campaigns to inform housing users of the benefits and options for the latter would be valuable if greater demand for such features is to be created.

## 6. Conclusions

The level of housebuilding in the UK needs to greatly increase in order to address the national housing shortage. However, while striving to deliver the estimated 250,000 to 300,000 new homes that are required each year and meet the environmental, particularly energy efficiency targets, it is important that the design of housing developments does not neglect the “softer” features that promote well-being of residents and the growth of sustainable communities.

This research focused on such “soft” features of housing and neighbourhood design that form a part of the sustainable and healthy housing framework, and sought to ascertain the level of importance that key housing stakeholders attach to them. The study identified significant differences between the opinions of these two groups of stakeholders for seven of the 11 “soft” features. In particular, the housing-user group attached a much greater level of importance to three features, namely “private outdoor space” (F2), “attractive views to outside” (F7), and “more compact neighbourhood” (F10) than did the housing providers. Conversely, housing providers regarded four features, specifically “dwelling adaptability” (F3), “opportunities to get involved” (F8), “security features” (F9), and “proximity to amenities” (F11) as more important than did the housing users. The opinion also differed among the three housing provider groups (local authorities, housing associations and private sector developers) for five of the 11 features, most notably with “dwelling adaptability” (F3) and “opportunities to get involved” (F8) being rated much higher by the social housing providers (local authorities and housing associations) than by the private sector developers.

This research has highlighted that the opinions of housing users and housing providers are not necessarily aligned with regards to the importance of certain “soft” features. A replication of the study and/or widening of the geographical scope would be valuable in revealing whether the same differences exist or the importance accorded to the each of the “soft” features is altered in different geographical areas (due to, for instance, cultural, climatic, socioeconomic factors). Furthermore, the study could be supplemented by a more qualitative approach to elucidate the reasons why certain stakeholders regard some features as more important than others. For instance, if housing provider opinions (where misaligned with those of housing users) are shaped by broader policies and best practice guidance, it would raise questions about whether these policies are in harmony with consumer needs and preferences.
